# Puppy Socialisation Experiences in Relation to Age and COVID-19 Lockdown Restrictions in the UK and ROI

**DOI:** 10.3390/ani14101471

**Published:** 2024-05-15

**Authors:** Rachel H. Kinsman, Hoi-Lam Jim, Rachel A. Casey, Eliza Ruiz-Izaguirre, Sara Owczarczak-Garstecka, Ben Cooper, Séverine Tasker, Jane K. Murray

**Affiliations:** 1 Dogs Trust, London EC1V 7RQ, UK; 2 Institute for Advanced Study, Kyoto University, Kyoto 6068501, Japan; 3 Japan Society for the Promotion of Science, Tokyo 1020083, Japan; 4 Bristol Veterinary School, University of Bristol, Bristol BS40 5DU, UK; 5 Linnaeus Veterinary Limited, Shirley, Solihull B90 4BN, UK

**Keywords:** dog, development, behaviour, human–dog relationship, coronavirus, lockdown, welfare

## Abstract

**Simple Summary:**

For dogs and their owners to live in harmony, owner expectations and the behaviour of their dogs need to be aligned. Limited socialisation can contribute to the development of undesirable behaviours, so the reduced socialisation opportunities for many puppies during the COVID-19 lockdown is a concern. This United Kingdom/Republic of Ireland study used data collected between May 2016 and November 2022 to examine the impact of age and lockdown phase (pre-, during, and post-) on the types of socialisation experiences of 8-to-19-week-old puppies and the recency of socialisation experiences of 6-month-old puppies. The findings showed that puppies under 19-weeks had more types of experiences as they aged, and during pre-lockdown compared to post-lockdown, but not between other lockdown phases. Most 6-month-old puppies had met new adults or dogs, familiar dogs, or children within the last 1–7 days, regardless of lockdown phase. However, during lockdown, 6-month-old puppies experienced longer periods between meeting new adults in their homes. Overall, lockdown had a smaller impact on socialisation experiences than expected, but the quantity and quality of these experiences may have been affected. Future research will explore whether these early-life experiences relate to adult behaviour as the dogs in the study grow older.

**Abstract:**

Limited socialisation can contribute to the development of undesirable dog behaviours. The COVID-19 lockdown potentially limited socialisation opportunities, which may negatively impact the future behaviour of puppies raised during lockdown. Data were gathered from longitudinal study participants in the United Kingdom/Republic of Ireland via multiple questionnaires between May 2016 and November 2022. The impact of age and lockdown phase (pre-, during, and post-) on the types of socialisation experiences of 8-to-19-week-old puppies and the recency of socialisation experiences of approximately 6-month-old puppies were examined. Puppies under 19-weeks had significantly more types of socialisation experiences (from a predefined list) as they aged, and pre-lockdown compared to post-lockdown, but not between other lockdown phases. Most 6-month-old puppies had met a new adult or dog outside the household, a familiar dog, and/or a child within the last 1–7 days, and this was similar between lockdown phases. During lockdown, 6-month-old puppies experienced longer periods between meeting a new adult in their home. Puppies were hypothesised to have had fewer experiences during lockdown, but this was not found. However, the quantity and quality of these experiences may have been affected. Future research within this longitudinal study will explore relationships between the timing and type of experiences had by puppies and their subsequent behaviour.

## 1. Introduction

Human–dog relationships are more successful when there is not too great a mismatch between owner expectations and dog behaviours. Undesirable behaviours have been widely reported to impact the welfare of the dog [1,2,3] and the wellbeing of the owner [4] and can lead to relinquishment [5,6,7,8,9], failed adoptions from rehoming organisations [10,11,12], and even euthanasia of healthy dogs [13,14,15,16]. Many factors are thought to play a role in the development of undesirable behaviours; one well-documented factor is early-life experiences including socialisation [17,18,19,20,21]. Thus, the focus of this study was to explore the socialisation experiences within a cohort of puppies participating in a longitudinal study.

Socialisation is a highly important developmental period for puppies that occurs from 3 weeks until 12–16 weeks of age, depending on the breed and individual [17,22,23]. During this period, puppies learn about their physical and social environment. To develop an ability to cope with novelty during adulthood, puppies should gradually be exposed to a variety of novel stimuli and environments at an early age [17,22,24]. Exposure to new situations should be at a level which leads to habituation (deceased responsiveness to a stimuli) rather than sensitisation (increased responsiveness to a stimuli) [25]. How a puppy responds to stimuli varies during the socialisation period, for example, Scott and Fuller [17] documented that puppies showed a higher tendency to approach new people and objects around 5 weeks of age, but subsequently, this tendency declines with age. Therefore, introductions to new stimuli should be adjusted to the level of development of the puppies and be at a level that does not cause avoidance to prevent overwhelming them [26,27]. Although dogs change behaviour throughout their lives, puppies are particularly sensitive to environmental influence and acquire specific responses and preferences more readily than at other periods of development during the socialisation period [28,29].

Most owners acquire a puppy after 8 weeks of age [30,31], and in many cases, breeders commence the process of habituating a puppy to novel stimuli [24]. Most existing research focuses on socialisation that occurs within the breeding environment or within clinical settings, as opposed to after the puppy has been acquired. Previous research has documented that reduced levels of socialisation can increase the probability of fearfulness towards unfamiliar people and dogs [20,21], as well as non-social fear, such as noise phobias [20,32,33] and fear of novel situations and surfaces [33]. Additionally, it has been reported that behaviours such as separation-related behaviour [27,34,35,36] and aggressive behaviour can be influenced by socialisation and the early-life environment [18,36,37]. Therefore, reduced levels of socialisation for young puppies are of great concern.

During the COVID-19 pandemic, a range of unprecedented restrictions were put into place in the United Kingdom (UK) and Republic of Ireland (ROI) starting from 23 March 2020, which included restrictions on exercise outside of the home, non-essential travel, visiting and meeting up with people from other households, and closure of non-essential shops and businesses. Also of relevance were restrictions on how veterinary practices operated and other dog-related services, such as puppy classes, dog walkers, and dog groomers. Due to these restrictions, it is possible that young puppies may have had reduced opportunities for socialisation. One study reported a significant increase in fearfulness and aggressive behaviour in dogs whose socialisation period overlapped with lockdown restrictions [38]. Using data collected retrospectively between 10 November and 31 December 2020, Brand et al. [39] compared the socialisation experiences of puppies under 16 weeks of age in the UK that were purchased between 23 March and 31 December 2019, (i.e., before the COVID-19 pandemic; *n* = 1148) and those purchased during the same dates in 2020 (i.e., during lockdown restrictions in the UK; *n* = 4369). They reported no significant reduction in the proportion of experiences that puppies purchased during 2020 had compared with those purchased in 2019. However, they did note reduced levels of puppy class attendance and visitors to the home during the critical developmental periods of the puppies purchased during the pandemic. Data from the present longitudinal study (“Generation Pup”, described later) differ from that of Brand et al. [39], as the data were collected prospectively for three time periods: pre-, during, and post-lockdown. Therefore, the study reported here can provide additional evidence related to this important topic.

As well as the potential for memory decay to impact the results of studies utilising retrospective data collection, owners’ knowledge and attitudes towards socialisation may have altered since their puppy went through socialisation, thus resulting in another potential mechanism for recall bias. For a topic such as socialisation, data collected prospectively have the advantage of decreasing the potential for recall bias. “Generation Pup” is a longitudinal study of many aspects of canine health and behaviour which, at time of publication, is still recruiting participants. Data are gathered via owner-completed questionnaires on topics such as the environment, family, health, behaviour, and experiences of dogs. Questionnaires are sent at various timepoints up until 18-months of age, after which point questionnaires are sent every six months [40].

To acquire a picture of the socialisation experiences “Generation Pup” owners provided for their puppies as they aged and during lockdown, this study sought to examine the owner-reported socialisation experiences of puppies at two age points: (1) between 8 and 19-weeks-old (i.e., 56–133 days); and (2) approximately 6-months-old. Two sets of questions were asked in separate questionnaires (i.e., were not asked at both timepoints so direct comparisons between the two timepoints could not be made). The first set of questions related to the types of socialisation experiences of puppies between 8 and 19-weeks-old (details of the ages brackets used are provided in the Section 2). The second set of questions related to the recency of socialisation experiences of 6-month-old puppies. Due to the nature of longitudinal studies, and as questionnaire completion was not compulsory, whether a dog was included in one or both timepoint datasets was dependent on whether their owner had completed both sets of questions in the questionnaires.

In addition to examining the type and recency of puppy socialisation experiences, the impact of COVID-19 lockdown restrictions in the UK and ROI on socialisation experiences was also investigated. This was due to the concerns about the limited levels of socialisation opportunities of puppies during the COVID-19 pandemic, and was possible as data were collected pre-, during, and post-lockdown restrictions. The objectives of this research were as follows:

- To summarise the different types of socialisation experiences (from a pre-defined list) of puppies as they aged to investigate how the proportion and type of experiences owners offered their puppies may change with age. The age categories were 56–77 days, 84–105 days, and 112–133 days.
- To determine whether the proportion of total types of socialisation experiences reported for puppies aged 56–133 days (inclusive) changed in relation to the lockdown phase to investigate whether lockdown resulted in puppies receiving limited socialisation during this time.
- To summarise the recency of socialisation experiences of 6-month-old puppies as reported by owners pre-, during, and post lockdown in the UK/ROI.
- To investigate the impact of lockdown phase on the proportion of recent socialisation experiences of 6-month-old puppies.

It was hypothesised that the types of socialisation experiences of puppies under 19-weeks-old would increase as they aged. This was because the experiences that were chosen were those that adult dogs might encounter on a regular basis. Therefore, to habituate puppies to these experiences, they should be gradually introduced to them from an early age [26,27]. It was predicted that puppies would have fewer experiences during lockdown compared to pre- and post-lockdown. However, an interaction effect between puppy age and lockdown phase was not anticipated, as it was predicted that puppies of all ages would be equally affected by lockdown. It was also predicted that 6-month-old puppies would experience longer durations between visitors to the home and reduced opportunities to meet new people and other dogs outside of the home during lockdown compared to pre- and post- lockdown.

## 2. Materials and Methods

### 2.1. Study Design and Participants

The inclusion criteria for “Generation Pup” were as follows: (1) participants must be resident in the UK or the ROI, (2) be at least 16 years old, and (3) own a puppy under 16-weeks-old at time of registration (or under 21 weeks if the puppy had been through quarantine). The study and recruitment methodology have been detailed in Murray et al. [40].

### 2.2. Data Collection

Data were gathered via self-administered questionnaires that were completed online or as postal questionnaires. Prior to analysis, all data were pseudonymised. As recruitment and data collection for “Generation Pup” were still ongoing at the time of this study, data from puppies registered with the project between 4 May 2016 and 24 November 2022 were used. Therefore, data were collected before, during, and after the COVID-19 lockdown. The date of questionnaire completion was used to categorise each questionnaire into one of three phases—pre-lockdown, lockdown, and post-lockdown—according to the timeline of UK government coronavirus lockdowns and restrictions [41,42]. The lockdown dates and restrictions, although fairly similar, varied not only between countries (i.e., England, Scotland, Wales, and Northern Ireland) but also between country regions (e.g., North East, South East, West Midlands, etc.). As most (over 75%) owners in “Generation Pup” were based in England, the phases were defined based on lockdown dates in England (i.e., the first lockdown began on 23 March 2020, and most legal limits on social contact were removed on 19 July 2021).

### 2.3. Inclusion Criteria for Analysis

The sample size was dependent on the number of owners who had completed three mandatory registration questionnaires (“About Me”, “About My Household”, and “About My Puppy”). Additionally, for inclusion in the analysis, owners needed to have completed one or more of the following questionnaires: the “Settling In” questionnaire (issued to owners 1–3 weeks after acquisition or until 12 weeks of age, whichever was sooner), the “12-week”, “16-week”, or “6-month” questionnaires (issued to owners when their puppies reached that age point and were available for completion for 24 days) [40].

### 2.4. Statistical Analysis of the Types of Socialisation Experiences of 8-to-19-Week-Old Puppies

The types of socialisation experiences of puppies and the effect of age was explored using questions included in the “Settling In”, “12-week”, and “16-week” questionnaires (for questions see Supplementary Materials). As mentioned, the critical socialisation period typically occurs between 3 and 12 weeks of age, but owners commonly do not acquire a puppy until 8 weeks of age in the UK [31]. Therefore, three distinct age categories (with a one-week gap between) were chosen to span the critical socialisation period and then to go beyond that period. These age groups were as follows: 56–77 days (8, up to 11 weeks), 84–105 days (12, up to 15 weeks), and 112–133 days (16, up to 19 weeks). For each completed questionnaire, the age of the puppy at questionnaire completion was determined, and if the age of the puppy was within one of these age categories, the data were included.

**Figure 1:**
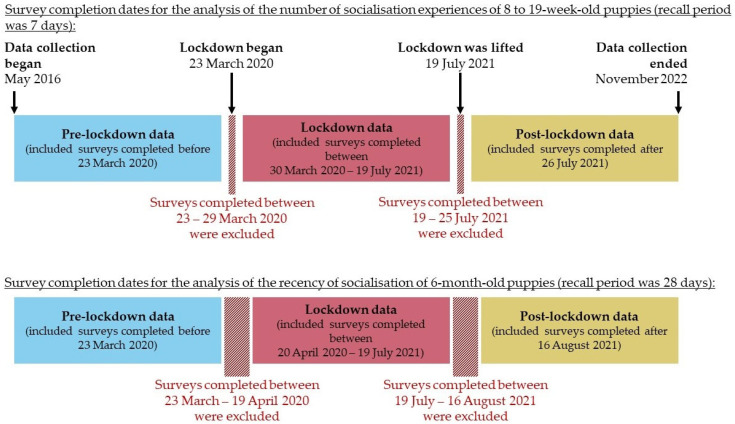
Definition of COVID-19 lockdown phases used in this study and the inclusion/exclusion criteria of questionnaires based on completion date.

To explore the impact of lockdown on the proportion of total types of experiences of puppies aged 56–77, 84–105, and 112–133 days, the lockdown phases defined in Figure 1 were used. As owners were asked to recall their puppy’s experiences in the past 7 days, data were excluded from questionnaires completed between 23 March 2020 and 29 March 2020 and between 19 July 2020 and 25 July 2020 to prevent data from an owner falling into more than one lockdown phase (i.e., if the dates of the recall period (“past 7 days”) spanned two lockdown phases). Likewise, the dates were adjusted, as shown in Figure 1, for data collected from the 6-month questionnaire to account for a 28-day recall period.

In each of the three questionnaires (“Settling In”, “12-week”, and “16-week”), owners were given a list of 16 experiences (Table 1) that their puppy may have had during the last 7 days and were asked to respond “yes”, “no”, or “I’m unsure” to indicate whether their puppy had had each experience. The proportion of puppies exposed to each experience type was determined for all 16 experiences across the three age groups and during each of the three lockdown phases. The owners’ responses to each of the 16 types of socialisation experiences were recoded into a binary variable (i.e., yes/no to 1/0); “I’m unsure” responses and missing data were excluded from analysis. The proportion of types of experiences each puppy had was calculated by adding the number of experience types the owner reported and dividing by the total number of potential experience types (i.e., 16 if the owner responded yes/no to all 16 experience types). Finally, the mean proportion of experience types for puppies in each age category was calculated.

All analyses were performed using R (v4.2.2 [43]) in RStudio (v2022.12.0.353 [44]), and graphs were created using the R package “ggplot2” (v.3.3.6 [45]). A generalised linear mixed-effects model (GLMM) with beta distribution using the R package “glmmTMB” (v1.1.5; [46]) was used to assess if age and the COVID-19 lockdown period affected the proportion of socialisation experiences. Power was calculated for this model by simulating full datasets equal in size to the collected data with fixed and random effect magnitudes and variances based off those observed. Data were repeatedly simulated with a range of different effect sizes, and to each was fitted a model with the same structure as the experimental beta GLMM. The proportion of simulations in which each effect size could be detected by these models at the *p* < 0.05 significance threshold was recorded, giving a minimum detectable effect size at the 80% power level of 0.039. This corresponds to a fold change in proportions of 1.04× or 0.96×. As a beta GLMM cannot be fitted when the response variable comprises 0 and 1, and the dataset comprised at least one 0, we transformed the response variable (the mean proportion of experiences) using the following formula (where *y* is the variable to be transformed and *N* is the sample size; see Smithson and Verkuilen [47]):

*y*″= [*y*′(*N* − 1) + 1/2]/*N*.

The test predictors were age (factor with three levels: 56–77, 84–195, and 112–133 days) and lockdown phase (factor with three levels: pre-lockdown, lockdown, and post-lockdown), and the model included an interaction between age and lockdown phase. As some owners completed questionnaires for their puppies at more than one age point and some owners had multiple dogs participating in the study, Dog ID and Owner ID were included in the model as random effects. Thus, the full model was as follows:

transformed mean proportion of experiences ~ age * lockdown phase + (1 | Dog ID) + (1 | Owner ID).

To avoid multiple testing [48], the significance of the full model (with all the fixed effects) was compared to a null model (lacking the test predictors but otherwise identical to the full model). This comparison was tested by means of a likelihood ratio test [49] using the R function “anova” with the argument test set to “Chisq”. Pairwise comparisons were conducted to evaluate the difference between levels using the R package “emmeans” (v1.8.4.1; [50]).

Model stability was assessed by comparing the estimates obtained from the model based on all data with those obtained from models with the levels of random effects excluded one at a time. This revealed the model to be extremely stable (see the range of estimates in Table S1), and confidence intervals were calculated using the function “boot.glmmTMB”. Collinearity was checked by determining the variance inflation factors (VIF) [51] for a standard linear model excluding the interaction and random effects using the function “vif” in the R package “car” (v3.1.1 [52]) for a standard linear model excluding interactions and random effects. This analysis revealed no problems of collinearity between the variables (VIF = 1.000), and the model was not overdispersed (dispersion parameter = 0.543). The R functions used for model stability, confidence intervals, and overdispersion were provided by Mundry [53].

### 2.5. Statistical Analysis of the Recency of Socialisation with People and Dogs Had by 6-Month-Old Puppies

To explore the recency of socialisation experiences of 6-month-old puppies with adults and other dogs, owners were asked approximately how many days it had been since their puppy had met any of the following (defined as the puppy having been in the same room and/or within approximately 3 m/10 feet) (for questions see Supplementary Materials):

- A new adult he/she did not previously know when they visited the household;
- A new adult he/she did not know when outside of the household;
- A new dog from outside of the household;
- A dog that he/she knows (defined as a dog that has been met at least twice before).

The available responses were as follows: “Today”, “2 days”, “3 days” … up to “28 days”, “More than 28 days”, “I don’t know/can’t remember”, and “Not applicable” (i.e., not met). The data indicated that there might have been some recall bias in reporting as it showed peaks at 7, 14, and 21 days. This potentially suggests that owners were estimating when their puppy had had the socialisation experience (i.e., within the last week, two weeks and so on). Thus, to minimise the potential for recall bias, the number of days since a 6-month-old puppy had had a socialisation experience was grouped into three categories: “1 to 7”, “8 or more”, and “not met”. “I don’t know/can’t remember” responses and missing data were excluded from analysis.

To explore whether the COVID-19 lockdown affected the recency of socialisation experiences of 6-month-old puppies, the data were again categorised into three phases: pre-lockdown, lockdown, and post-lockdown. As the highest number of days an owner could select was “More than 28 days” for these questions, 28 days was used to define the differences between lockdown phases. Thus, data were excluded from questionnaires completed between 23 March and 19 April 2020 and 19 July and 15 August 2021 (Figure 1).

Additionally, the recency of socialisation experiences of 6-month-old puppies with children were investigated. Owners were asked if the puppy had met (defined as interacted with or been aware of whilst in close proximity to) a baby, a toddler to 4-year-old child, a child aged 5–10 years, a child aged 11–15 years, or a child of unknown age in the last two months (excluding children living in their household). If the puppy had met a child, the owner was asked approximately how many days had passed since the meeting. The available responses, exclusion criteria, and the categorisation of “days” were the same as above. All the child categories were grouped together; to determine the number of days since the last meeting with a child, the lowest number of days reported in any category was included in the analysis. For example, if a puppy had never met a baby but had met a toddler 8 or more days ago and a child aged 11–15 years 1 to 7 days ago, the response was categorised as “1 to 7” days.

A generalised linear model (GLM) with binomial distribution was conducted using the function “glm” for each of the five socialisation experiences:

1. A new adult the puppy did not previously know when they visited the household;
2. A new adult the puppy did not know when outside of the household;
3. A new dog from outside of the household;
4. A dog that the puppy knows;
5. A child/children.

To determine the power for this model, 1,000,000 random simulations of a 2 × 3 contingency table of 3275 individuals were performed, and to each was fitted a model with a structure identical to the experimental binomial GLM. Following the same process as the beta GLMM, a minimum detectable effect size at the 80% power level of 0.272 was determined, equivalent to a fold change in proportions of 1.312× or 0.761×.

The test predictors in the model were days (factor with three levels: 1 to 7, 8 or more, and not met) and lockdown phase (factor with three levels: pre-lockdown, lockdown, and post-lockdown). The model included an interaction between lockdown phase and days, and the response variable was the proportion of dogs in each lockdown phase and category of “days” since the socialisation experience in question. Thus, the full model was as follows:

proportion of dogs ~ lockdown phase * days.

## 3. Results

When this study commenced, 6809 puppies had been registered with “Generation Pup”, and recruitment was still ongoing. After data cleaning, 894 puppies (13.1% of the sample of registered puppies) were removed due to non-completion of one or more of the questionnaires of interest; therefore, the analysis presented here used data from 5915 puppies. For detailed demographic data of the first 3726 owners and puppies registered on the “Generation Pup” project, please see Murray et al. [40].

### 3.1. Types of Socialisation Experiences of 8-to-19-Week-Old Puppies

**Figure 2:**
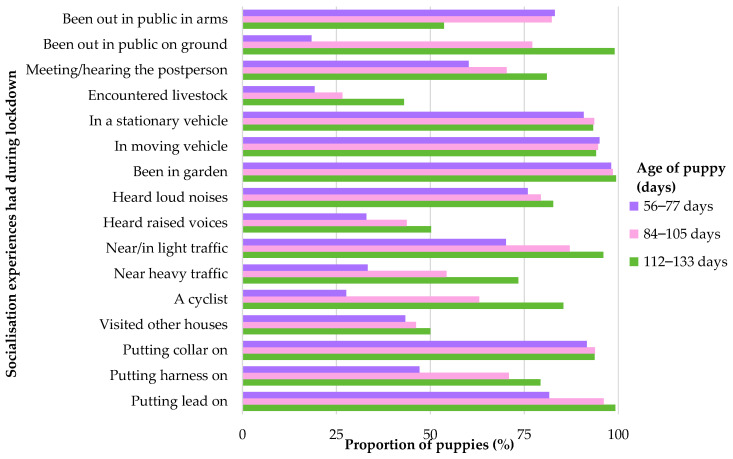
The proportion of puppies aged 56–77, 84–105, and 112–133 days that had had these socialisation experiences during a 7-day period during the COVID-19 lockdown.

Of the 5915 puppies with available data, there were 2069 completed “Settling In” questionnaires, 3680 completed “12-week” questionnaires, and 4111 completed “16-week” questionnaires. However, puppies that were outside of the age criterion described in Section 2.2 and data collected in the overlap periods between lockdown phases (Figure 1) were removed from the analysis (504, 224, and 190 puppies from the “Settling In”, “12-week”, and “16-week” questionnaires, respectively). Therefore, the final sample sizes were 1565 puppies aged 56–77 days, 3456 puppies aged 84–105 days, and 3921 aged 112–133 days. Figure 2 shows the proportion of puppies that had had the listed types of socialisation experiences in the past 7 days, as reported by their owners during the COVID-19 lockdown. Table 2 summarises the number of different types of experiences of puppies (during the previous 7 days) in the three age categories pre-, during, and post-lockdown.

The likelihood ratio test comparing the full and null model revealed that the interaction between age and lockdown phase was non-significant (χ^2^ = 3.544, *df* = 4, *p* = 0.471). Therefore, the interaction term was dropped from the full model to examine the reduced model (containing only the main effects). The reduced model revealed that the test predictors were significantly associated with the outcome (age: χ^2^ = 2058.365, *df* = 2, *p* < 0.001; lockdown phase: χ^2^ = 8.935, *df* = 2, *p* = 0.011; Table S1 in Supplementary Materials).

**Figure 3:**
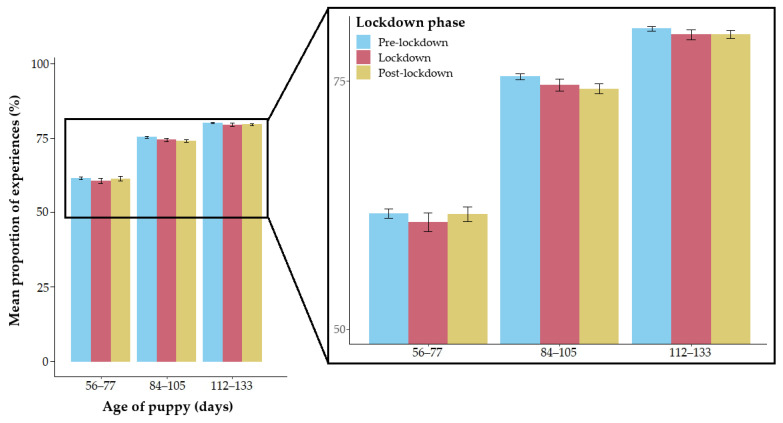
The mean proportion of types of socialisation experiences of puppies aged 56–77, 84–105, and 112–133 days. Error bars indicate standard errors of the mean.

The pairwise comparisons revealed that there were significant differences between all ages (Table 3). Specifically, puppies had a higher proportion of experience types at age 84–105 days and at age 112–133 days compared to at age 56–77 days (Figure 3). Additionally, puppies aged 112–133 days had a higher proportion of experiences (from the list of 16) compared to puppies aged 84–105 days (all *p* < 0.001) (Figure 3). Thus, puppies were reported to have had more types of experiences during the previous 7 days as they grew older. Also, puppies had a significantly higher proportion of different experience types pre-lockdown compared to post-lockdown (*p* = 0.020), but there was no significant difference between pre-lockdown vs. lockdown (*p* = 0.132) or lockdown vs. post-lockdown (*p* = 0.944) (Table 3, Figure 3).

### 3.2. The Recency of Socialisation with People and Dogs Had by 6-Month-Old Puppies

Data were available from 3360 completed “6-month” questionnaires. However, data collected in the two overlap periods between lockdown phases (Figure 1) were removed (60 and 25 puppies, respectively). Therefore, the final sample size was 3275 6-month-old puppies.

#### 3.2.1. Puppies That Had Met a New Adult Who Visited Their Household

**Figure 4:**
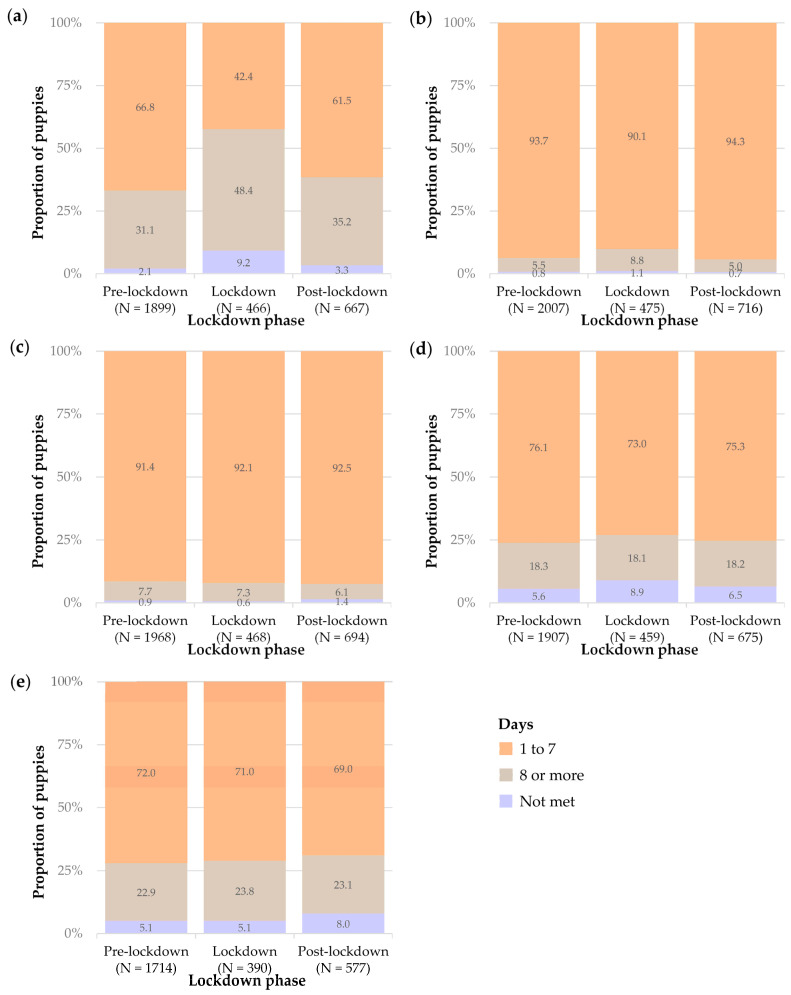
Stacked bar charts showing the percentage (and number) of dogs in each lockdown phase for the recency of socialisation experiences of 6-month-old puppies with (**a**) a new adult when they visited the household; (**b**) a new adult met when outside of the household; (**c**) a new dog from outside of the household; (**d**) a dog that the puppy knows; (**e**) a child/children.

There was a significant interaction between lockdown phase and days (χ^2^ = 177.99, *df* = 4, *p* < 0.001, Table S2 in Supplementary Materials). The pairwise comparisons showed that there were significant differences between all categories of days: specifically, a large proportion of puppies had met a new adult who visited their household between 1 to 7 days ago, a significantly smaller proportion of puppies had met new adults visiting their households 8 or more days ago, and an even smaller proportion of puppies had not met a new adult who visited their household. This pattern of proportions was observed in the pre-lockdown and post-lockdown phases (all *p* < 0.001). However, there was a different pattern during lockdown: namely, there was no significant difference between the proportion of puppies meeting new adult visitors to the household 1 to 7 days prior to questionnaire completion and 8 or more days prior to questionnaire completion (*p* = 0.165). These results indicate that new adults visited the households of puppies less frequently during lockdown compared to pre- or post-lockdown (Table 4, Figure 4a).

#### 3.2.2. Puppies That Had Met a New Adult When Outside of the Household

A significant interaction between lockdown phase and days was found (χ^2^ = 16.989, *df* = 4, *p* < 0.002, Table S3 in Supplementary Materials). The pairwise comparisons showed a significant difference in all categories of days for all lockdown phases (Table 4, Figure 4b). The majority of puppies had met a new adult when outside of the household recently (within the last 1 to 7 days). A significantly smaller proportion of puppies had met a new adult when outside of the household a longer time ago (8 or more days), and an even smaller proportion had not met a new adult when outside of the household at 6 months old. These differences were significant between pre-, during, and post-lockdown (all *p* < 0.001).

#### 3.2.3. Puppies That Had Met a New Dog from Outside of the Household and Puppies That Had Met a Familiar Dog

No significant interactions were found between lockdown phase and days since meeting a dog from outside of the household or in the model for days since meeting a familiar dog (new dog: χ^2^ = 5.338, *df* = 4, *p* = 0.254, familiar dog: χ^2^ = 8.369, *df* = 4, *p* = 0.079; Tables S4 and S5 in Supplementary Materials). Thus, the test predictors were inspected independently. In both models, there was a significant difference between the categories of days. Again, most puppies had met a new/familiar dog within the last 1 to 7 days. A significantly smaller proportion of puppies had met a new/familiar dog 8 or more days ago, and an even smaller proportion of puppies had not met a new dog from outside of the household or a familiar dog (all *p* < 0.001). The pattern of responses did not significantly differ between lockdown phases (Table 5, Figure 4c,d).

#### 3.2.4. Puppies That Had Met a Child/Children

No significant interaction was found between lockdown phase and days since meeting a child/children (likelihood ratio test: χ^2^ = 8.552, *df* = 4, *p* = 0.073, Table S6 in Supplementary Materials). Thus, the test predictors were inspected independently. Again, there was a significant difference between the categories of days, which followed the same pattern as above (i.e., most puppies had met a child/children between “1 to 7” days, followed by “8 or more” days, followed by “not met”) (all *p* < 0.001), and there was no significant difference between lockdown phases (Table 5, Figure 4e).

## 4. Discussion

The future welfare of puppies who may have had reduced levels of socialisation experiences during the COVID-19 lockdown is a concern. Thus, this study investigated the types of socialisation experiences of 8-to-19-week-old puppies as reported by their owners pre-, during, and post-lockdown. As predicted, puppies were reported to have been exposed to significantly more types of experiences (from the list of 16) during the previous 7 days as they grew older. This was hypothesised because it was expected that owners, understanding the importance of socialisation, would take steps to provide different experiences for their puppies, which would be potentially built up gradually over time as their puppy aged. Additionally, it is recommended that puppies are given a primary vaccination course at approximately 8–10 weeks of age with follow-up doses around 2–5 weeks later [54,55]. Therefore, most puppies over approximately 16 weeks of age should have completed vaccinations and been allowed to go out in public on the ground. It follows that this would lead to a decrease with age in the proportion of puppies out in public in arms, which was indeed what was observed, but even at the older age timepoint, over 50% of puppies were still being carried. Despite this observed decrease with age for one of the socialisation variables, the overall finding that puppies had more types of experiences as they grew older was still significant.

Contrary to the second hypothesis that puppies would have had fewer experiences (from the list of 16) “in the last 7 days” during lockdown, puppies were only found to have a slightly but significantly higher proportion of experiences pre-lockdown compared to post-lockdown. Whilst this effect was significant, the effect size was small, indicating that the relationship is subtle. No significant difference between pre-lockdown vs. lockdown or lockdown vs. post-lockdown was found; thus, puppies in the “Generation Pup” cohort were not reported to have had a lower proportion of experiences (detailed in the questionnaire) during lockdown. These findings are in line with Brand et al. [39], which reported no significant reduction in the proportion of socialisation experiences of puppies under 16 weeks that were purchased during the pandemic, compared to those purchased in 2019.

Based on the measures of socialisation and results of both this current study and Brand et al. [39], lockdown does not appear to have significantly impacted puppy socialisation experiences (or at least those listed in Table 1). However, it is important to note that the “Generation Pup” questionnaires were created in 2015 and were not designed specifically for data collection during a pandemic, wherein such unprecedented restrictions occurred. Many socialisation experiences may have continued despite lockdown, but factors that potentially could have been impacted by lockdown, and which were not captured here, include visits to veterinary practices and dog groomers. Also, meeting new people, meeting new dogs, and people visiting the house (excluding “meeting/hearing the post person”) were not explored explicitly for 8-to-19-week-old puppies and were only examined in detail for 6-month-old puppies. One experience that would be hypothesised to be impacted considerably by lockdown was “visiting other houses”. The proportion of puppies that had visited other houses was lowest during lockdown for puppies in all three age categories (Table 2). If the list of 16 experiences had included more experiences that were centred around exposure to people and animals, the differences between lockdown phases observed might have been larger.

Additionally, during the most stringent restrictions (March–May 2020), people were only permitted to leave home for essential purposes, such as buying food or medication; thus, visiting other households was not allowed during this time [56]. However, as the lockdown phases used in the two analyses (30 March 2020–19 July 2021 and 20 April 2020–19 July 2021) spanned a period where lockdown restrictions varied and were eased and reinstated, the proportions reported are perhaps not as low as would have been expected if a shorter lockdown phase with more rigid restrictions had been used for the analyses. This is speculated as the date of birth of the puppy in relation to lockdown will likely have resulted in some puppies having fewer experiences of visiting other households. Additionally, the lockdown dates used in this study were those implemented in England. Roughly a quarter of dogs in the dataset lived in Scotland, Wales, Northern Ireland, or ROI; although the lockdown dates were similar between the four countries, it is plausible that this factor may have had some effect. It is not known whether all owners chose to adhere to the lockdown restrictions, as the questionnaires did not include questions on lockdown compliance. However, previous studies of the public documented poor adherence to self-isolation and social distancing restrictions [57,58], which could also have contributed to the lack of significant differences in the recency of socialisation experiences of 6-month-old puppies between the lockdown phases.

Another potential explanation for the observations not being as hypothesised comes from the findings of Holland et al. [59]. They documented a reduction in dogs’ exposure to people, other dogs, and everyday phenomena (e.g., traffic noise, and car journeys) during the pandemic, but also stated that many owners were concerned and aware of the importance of socialisation and, as they were unable to achieve this during lockdown, compensated by focusing more on dog training [59]. This is a feasible explanation in the “Generation Pup” study due to potential sampling bias of self-selection by committed dog owners [40]. Thus, if “Generation Pup” owners compensated for reduced levels of socialisation with other dogs and people by boosting exposure to experiences within the list of 16, it is speculated that this could be one potential explanation for the lack of difference between the proportions of experiences during lockdown compared to pre- and post-lockdown. For example, spending time in the garden and exposing the puppy to loud noises have the highest reported proportions during lockdown of the three timepoints. Also, meeting a cyclist had the highest proportion reported during lockdown, which potentially reflects the increase in popularity of cycling that occurred during lockdown [60].

The second part of the study focused on the recency of socialisation experiences of 6-month-old puppies with people and other dogs to investigate other aspects of socialisation that were not captured with the list of 16 socialisation experiences. By and large, the results revealed a similar pattern in the owners’ responses for all socialisation experiences: most puppies had had the experiences between 1 to 7 days previously, a significantly smaller proportion of puppies had had the experiences 8 or more days ago, and an even smaller proportion of puppies had never had these experiences. These responses did not differ between the lockdown phases for meeting a new dog from outside of the household, meeting a familiar dog, or meeting a child, and are discussed later. There were two exceptions: The first and most important distinction in this pattern was that during lockdown, most puppies had met a new adult when they visited the household 8 or more days ago, rather than within the last 1 to 7 days. In fact, there was no significant difference between the proportion of puppies that had met a new adult who visited their household between 1 to 7 days ago and 8 or more days ago during lockdown. Figure 4a indicates that puppies experienced longer periods between meeting a new adult inside their household during lockdown compared to pre- and post-lockdown. This finding is despite the previous discussion points about the duration and the fluctuation of restrictions during the lockdown phase used in this study. Nevertheless, this makes sense since people were not allowed to visit other households during lockdown, and the results are in line with Brand et al. [39], which reported that puppies under 16 weeks (similar in age to those in the present study) purchased during the pandemic were significantly less likely to have had visitors to their household compared to puppies purchased during 2019.

The other exception was seen in the proportion of puppies that met a new adult when outside of the household. The response in the categories of days (“1 to 7”, “8 or more”, and “not met”) were all significantly different to each other in all lockdown phases for puppies that had met a new adult when outside of the household. However, Figure 4b illustrates that the proportion of puppies in this model does in fact resemble the same pattern as the main finding (i.e., most puppies had had the experience between 1 to 7 days ago, a significantly smaller proportion of puppies had had the experience 8 or more days ago, and an even smaller proportion of puppies had never had the experience, and these responses are similar between the lockdown phases). The differences may have been statistically significant because of the large sample size, which allows for the detection of even small differences, so the applied significance of this finding should be interpreted carefully.

Overall, the results indicate that a large proportion of 6-month-old puppies had met a new adult or dog from outside of the household, a familiar dog, and a child within the last 1 to 7 days compared to 8 or more days or not met, and this was similar between all lockdown phases. A potential explanation for these findings is that owners may have continued to walk their puppies during lockdown regularly and thus had the same opportunities for social encounters as pre- and post-lockdown; although there were restrictions on exercise during lockdown, the government guidelines did not include clear limitations on dog walking [61]. However, it is important to note that this is speculative, as the questionnaire did not include questions about the owners’ dog walking behaviours specifically in relation to COVID-19 restrictions. Previous research has documented that owners’ dog walking behaviour and possibilities for social encounters did change during lockdown. For example, one study reported a significant reduction in the number of walks per day during lockdown (referring to during May 2020) compared to pre-lockdown (referring to during early/mid-February 2020), and a significant but less marked decrease in daily walk duration [62]. Another study reported that owners walked their dogs less frequently but for longer durations during lockdown (referring to May/June 2020), so the total duration of time spent walking per week remained similar [63]. Although interactions with other dogs reportedly decreased [62] and some owners felt the walks inadequately met their dog’s exercise and social interaction requirements during lockdown [59], people were permitted to meet outside in groups of up to six people [56] from 1 June 2020 (i.e., during part of the lockdown phase in this study). Thus, it is plausible that dog owners could have arranged to walk their puppies with other adults, their children, and their dogs, which may explain why there was no significant difference in the comparisons of lockdown phases for the socialisation experiences of meeting a new or familiar dog. As for exposure to children not being impacted by lockdown, it was speculated that dogs may have encountered more children during lockdown compared to pre- and post-lockdown as many schools were closed during a considerable period of the lockdown. However, there was no significant difference between lockdown phases and socialisation experiences with children.

A limitation of this study is that owners were asked to report which experiences their puppy had had, but the quantity and quality of each experience was not recorded. For example, the number of new or familiar people, children, and dogs that the puppy had met was not asked, nor the level of interaction the person/child/dog had with the puppy. The puppy’s behavioural response to each experience was recorded by owners in the questionnaire but not analysed in this study; this will be analysed in a future study to comprehend whether the exposure was a positive or negative experience for the puppy. Furthermore, there are a few limitations regarding the owners participating in “Generation Pup”. First, as data were collected prospectively and at repeated timepoints, it is possible that owners may have anticipated being asked about their puppies’ socialisation experiences, especially after encountering these questions for the first time, and may have made a concerted effort to expose their puppy to novel things. Second, longitudinal studies require multiple questionnaires to be completed over time, which can lead to self-selection bias and under-representation of lower socioeconomic backgrounds [64]. Additionally, only dogs with completed questionnaires were included in the analysis. This could potentially have introduced bias due to loss to follow-up if owners who partially completed a questionnaire or withdrew from the study implemented different socialisation practices. Third, most respondents are female (89.7% as reported by Murray et al. [40]), but typically, females are over-represented in human–pet relationships studies [65,66,67]. If socioeconomic background and the gender of the respondent influenced compliance with lockdown restrictions, the findings presented here might not reflect that of the wider dog-owning population in the UK/ROI.

## 5. Conclusions

This study has provided an insight into the types of socialisation experiences of puppies before, during, and after an unprecedented lockdown. The results revealed that the COVID-19 lockdown in the UK/ROI had a smaller impact on the socialisation experiences of “Generation Pup” puppies than hypothesised. Although the lockdown did not seem to have impacted the proportion of socialisation experiences, the quantity and quality of these experiences may have been affected. The long-term impacts, particularly for the puppies who had their socialisation period during lockdown, should still be explored in future research due to the potential consequences on adult behaviour. The longitudinal nature of “Generation Pup” will allow for the relationships between the timing and type of socialisation experiences had by puppies and their subsequent behaviour as adults to be explored as the dogs in the cohort age. A primary outcome of interest will be stranger-related aggression or fear, particularly in the context of strangers visiting the household.

## Figures and Tables

**Table 1:** List of experiences that owners were asked to report. Puppy must have had the encounter (defined as having experienced/been aware of the situation/person/animals in the question) during the last 7 days prior to completion of the “Settling In”, “12-week”, and “16-week” questionnaires.

Experiences
Being out in public on the ground
Being out in public in arms
Meeting/hearing the postperson
Encountering livestock
Being put in a stationary car or other vehicle
Travelling in a moving car or other vehicle
Going in the garden/yard
Hearing loud noises (e.g., bangs, fireworks etc.)
Hearing raised voices or arguments in the house
Being near light traffic (e.g., cars passing, quiet road), except when inside a vehicle
Being near heavy traffic (e.g., lorries, trucks passing, busy road), except when inside a vehicle
Encountering a person riding a bicycle
Visiting other houses than the one he/she lives in
Having a collar put on
Having a harness put on
Having a lead put on

**Table 2:** Summary of experiences for puppies aged 56–77 days, 84–105 days, and 112–133 days pre, during, and post-COVID-19 lockdown.

		Lockdown Phase								
		Pre-Lockdown (n = 959) n (%)			Lockdown (n = 261) n (%)			Post-Lockdown (n = 345) n (%)		
Age	Experienced in the Past 7 Days	Yes	No	Unsure ^1^	Yes	No	Unsure ^1^	Yes	No	Unsure ^1^
56–77 days (N = 1565)	Been out in public in arms	872 (90.9)	79 (8.2)	8 (0.8)	217 (83.1)	43 (16.5)	1 (0.4)	307 (89.0)	34 (9.9)	4 (1.2)
	Been out in public on ground	184 (19.2)	764 (79.7)	11 (1.1)	48 (18.4)	210 (80.5)	3 (1.1)	42 (12.2)	299 (86.7)	4 (1.2)
	Meeting/hearing the postperson	585 (61.0)	332 (34.6)	42 (4.4)	157 (60.2)	97 (37.2)	7 (2.7)	216 (62.6)	110 (31.9)	19 (5.5)
	Encountered livestock	176 (18.4)	770 (80.3)	13 (1.4)	50 (19.2)	208 (79.7)	3 (1.1)	57 (16.5)	281 (81.4)	7 (2.0)
	In a stationary vehicle	894 (93.2)	56 (5.8)	9 (0.9)	237 (90.8)	23 (8.8)	1 (0.4)	320 (92.8)	21 (6.1)	4 (1.2)
	In a moving vehicle	935 (97.5)	16 (1.7)	8 (0.8)	248 (95.0)	12 (4.6)	1 (0.4)	328 (95.1)	13 (3.8)	4 (1.2)
	Been in garden	930 (97.0)	22 (2.3)	7 (0.7)	256 (98.1)	4 (1.5)	1 (0.4)	333 (96.5)	8 (2.3)	4 (1.2)
	Heard loud noises	686 (71.5)	245 (25.5)	28 (2.9)	198 (75.9)	57 (21.8)	6 (2.3)	239 (69.3)	93 (27.0)	13 (3.8)
	Heard raised voices	353 (36.8)	577 (60.2)	29 (3.0)	86 (33.0)	168 (64.4)	7 (2.7)	117 (33.9)	220 (63.8)	8 (2.3)
	Near/in light traffic	675 (70.4)	272 (28.4)	12 (1.3)	183 (70.1)	77 (29.5)	1 (0.4)	259 (75.1)	79 (22.9)	7 (2.0)
	Near heavy traffic	338 (35.2)	608 (63.4)	13 (1.4)	87 (33.3)	171 (65.5)	3 (1.1)	130 (37.7)	208 (60.3)	7 (2.0)
	A cyclist	205 (21.4)	718 (74.9)	36 (3.8)	72 (27.6)	178 (68.2)	11 (4.2)	87 (25.2)	244 (70.7)	14 (4.1)
	Visited other houses	498 (51.9)	452 (47.1)	9 (0.9)	113 (43.3)	147 (56.3)	1 (0.4)	154 (44.6)	187 (54.2)	4 (1.2)
	Putting collar on	869 (90.6)	83 (8.7)	7 (0.7)	239 (91.6)	21 (8.0)	1 (0.4)	313 (90.7)	28 (8.1)	4 (1.2)
	Putting harness on	434 (45.3)	517 (53.9)	8 (0.8)	123 (47.1)	137 (52.5)	1 (0.4)	189 (54.8)	152 (44.1)	4 (1.2)
	Putting lead on	740 (77.2)	212 (22.1)	7 (0.7)	213 (81.6)	47 (18.0)	1 (0.4)	262 (75.9)	79 (22.9)	4 (1.2)
		**Pre-Lockdown (n = 2154)**			**Lockdown (n = 519)**			**Post-Lockdown (n = 783)**		
**Age**	**Experienced in the Past 7 Days**	**Yes**	**No**	**Unsure ^1^**	**Yes**	**No**	**Unsure ^1^**	**Yes**	**No**	**Unsure ^1^**
84–105 days (N = 3456)	Been out in public in arms	1841 (85.5)	304 (14.1)	9 (0.4)	427 (82.3)	86 (16.6)	6 (1.2)	665 (84.9)	113 (14.4)	5 (0.6)
	Been out in public on ground	1676 (77.8)	468 (21.7)	10 (0.5)	400 (77.1)	112 (21.6)	7 (1.3)	558 (71.3)	217 (27.7)	8 (1.0)
	Meeting/hearing the postperson	1489 (69.1)	552 (25.6)	113 (5.2)	365 (70.3)	130 (25.0)	24 (4.6)	527 (67.3)	199 (25.4)	57 (7.3)
	Encountered livestock	655 (30.4)	1464 (68.0)	35 (1.6)	138 (26.6)	373 (71.9)	8 (1.5)	206 (26.3)	567 (72.4)	10 (1.3)
	In a stationary vehicle	2071 (96.1)	75 (3.5)	8 (0.4)	486 (93.6)	27 (5.2)	6 (1.2)	733 (93.6)	45 (5.7)	5 (0.6)
	In a moving vehicle	2103 (97.6)	44 (2.0)	7 (0.3)	491 (94.6)	22 (4.2)	6 (1.2)	748 (95.5)	30 (3.8)	5 (0.6)
	Been in garden	2121 (98.5)	26 (1.2)	7 (0.3)	511 (98.5)	2 (0.4)	6 (1.2)	764 (97.6)	13 (1.7)	6 (0.8)
	Heard loud noises	1673 (77.7)	385 (17.9)	96 (4.5)	412 (79.4)	83 (16)	24 (4.6)	587 (75.0)	162 (20.7)	34 (4.3)
	Heard raised voices	1057 (49.1)	1038 (48.2)	59 (2.7)	227 (43.7)	271 (52.2)	21 (4.0)	328 (41.9)	430 (54.9)	25 (3.2)
	Near/in light traffic	1875 (87.0)	267 (12.4)	12 (0.6)	452 (87.1)	60 (11.6)	7 (1.3)	687 (87.7)	86 (11)	10 (1.3)
	Near heavy traffic	1253 (58.2)	864 (40.1)	37 (1.7)	282 (54.3)	229 (44.1)	8 (1.5)	440 (56.2)	329 (42)	14 (1.8)
	A cyclist	1186 (55.1)	864 (40.1)	104 (4.8)	327 (63.0)	162 (31.2)	30 (5.8)	410 (52.4)	342 (43.7)	31 (4)
	Visited other houses	1421 (66.0)	723 (33.6)	10 (0.5)	240 (46.2)	272 (52.4)	7 (1.3)	480 (61.3)	297 (37.9)	6 (0.8)
	Putting collar on	2025 (94.0)	121 (5.6)	8 (0.4)	487 (93.8)	26 (5.0)	6 (1.2)	732 (93.5)	45 (5.7)	6 (0.8)
	Putting harness on	1361 (63.2)	785 (36.4)	8 (0.4)	368 (70.9)	145 (27.9)	6 (1.2)	610 (77.9)	167 (21.3)	6 (0.8)
	Putting lead on	2075 (96.3)	71 (3.3)	8 (0.4)	499 (96.1)	14 (2.7)	6 (1.2)	754 (96.3)	24 (3.1)	5 (0.6)
		**Pre-Lockdown (n = 2506)** **n (%)**			**Lockdown (n = 526)** **n (%)**			**Post-Lockdown (n = 889)** **n (%)**		
**Age**	**Experienced in the Past 7 Days**	**Yes**	**No**	**Unsure ^1^**	**Yes**	**No**	**Unsure ^1^**	**Yes**	**No**	**Unsure ^1^**
112–133 days (N = 3921)	Been out in public in arms	1428 (57.0)	1063 (42.4)	15 (0.6)	282 (53.6)	244 (46.4)	0 (0.0)	511 (57.5)	371 (41.7)	7 (0.8)
	Been out in public on ground	2443 (97.5)	47 (1.9)	16 (0.6)	521 (99.0)	5 (1.0)	0 (0.0)	859 (96.6)	24 (2.7)	6 (0.7)
	Meeting/hearing the postperson	1905 (76.0)	476 (19)	125 (5.0)	426 (81.0)	85 (16.2)	15 (2.9)	698 (78.5)	150 (16.9)	41 (4.6)
	Encountered livestock	1139 (45.5)	1318 (52.6)	49 (2.0)	226 (43.0)	295 (56.1)	5 (1.0)	339 (38.1)	530 (59.6)	20 (2.2)
	In a stationary vehicle	2407 (96.0)	88 (3.5)	11 (0.4)	491 (93.3)	35 (6.7)	0 (0.0)	849 (95.5)	34 (3.8)	6 (0.7)
	In a moving vehicle	2451 (97.8)	45 (1.8)	10 (0.4)	495 (94.1)	31 (5.9)	0 (0.0)	860 (96.7)	23 (2.6)	6 (0.7)
	Been in garden	2485 (99.2)	11 (0.4)	10 (0.4)	523 (99.4)	3 (0.6)	0 (0.0)	875 (98.4)	8 (0.9)	6 (0.7)
	Heard loud noises	1948 (77.7)	444 (17.7)	114 (4.5)	435 (82.7)	76 (14.4)	15 (2.9)	700 (78.7)	148 (16.6)	41 (4.6)
	Heard raised voices	1252 (50.0)	1184 (47.2)	70 (2.8)	264 (50.2)	251 (47.7)	11 (2.1)	365 (41.1)	490 (55.1)	34 (3.8)
	Near/in light traffic	2405 (96.0)	85 (3.4)	16 (0.6)	505 (96.0)	21 (4.0)	0 (0.0)	844 (94.9)	39 (4.4)	6 (0.7)
	Near heavy traffic	1823 (72.7)	650 (25.9)	33 (1.3)	386 (73.4)	139 (26.4)	1 (0.2)	631 (71.0)	245 (27.6)	13 (1.5)
	A cyclist	1923 (76.7)	472 (18.8)	111 (4.4)	449 (85.4)	68 (12.9)	9 (1.7)	668 (75.1)	176 (19.8)	45 (5.1)
	Visited other houses	1767 (70.5)	718 (28.7)	21 (0.8)	263 (50.0)	263 (50.0)	0 (0.0)	601 (67.6)	279 (31.4)	9 (1.0)
	Putting collar on	2334 (93.1)	160 (6.4)	12 (0.5)	493 (93.7)	33 (6.3)	0 (0.0)	824 (92.7)	59 (6.6)	6 (0.7)
	Putting harness on	1864 (74.4)	631 (25.2)	11 (0.4)	417 (79.3)	109 (20.7)	0 (0.0)	743 (83.6)	140 (15.7)	6 (0.7)
	Putting lead on	2478 (98.9)	17 (0.7)	11 (0.4)	522 (99.2)	4 (0.8)	0 (0.0)	875 (98.4)	8 (0.9)	6 (0.7)

^1^ Excluded from the analysis.

**Table 3:** Pairwise comparisons for the reduced beta regression model on the types of socialisation experiences of puppies aged 56–77, 84–105, and 112–133 days.

Term	Comparisons	Estimate	*SE*	*p*	95% CI	
					Lower	Upper
Age of puppy	56–77 days vs. 84–105 days	−0.736	0.021	<0.001	−0.785	−0.686
	56–77 days vs. 112–133 days	−1.072	0.022	<0.001	−1.123	−1.021
	84–105 days vs. 112–133 days	−0.336	0.017	<0.001	−0.376	−0.296
Lockdown phase	Pre-lockdown vs. Lockdown	0.059	0.031	0.132	−0.013	0.131
	Pre-lockdown vs. Post-lockdown	0.070	0.026	0.020	0.009	0.132
	Lockdown vs. Post-lockdown	0.011	0.035	0.944	−0.071	0.094

**Table 4:** Pairwise comparisons within the interaction in the binomial regression models for the recency socialisation experiences of 6-month-old puppies with adults inside and outside of the household.

Socialisation Experience	Lockdown Phase	Comparisons	Estimate	*SE*	*p*	95% CI	
						Lower	Upper
A new adult met when they visited the household (Figure 4a)	Pre-lockdown	1 to 7 days vs. 8 or more days	1.492	0.070	<0.001	1.330	1.660
		1 to 7 days vs. not met	4.537	0.167	<0.001	4.150	4.930
		8 or more days vs. not met	3.044	0.167	<0.001	2.650	3.440
	Lockdown	1 to 7 days vs. 8 or more days	−0.245	0.135	0.165	−0.560	0.070
		1 to 7 days vs. not met	1.983	0.190	<0.001	1.540	2.430
		8 or more days vs. not met	2.228	0.189	<0.001	1.780	2.670
	Post-lockdown	1 to 7 days vs. 8 or more days	1.076	0.114	<0.001	0.810	1.340
		1 to 7 days vs. not met	3.845	0.231	<0.001	3.300	4.390
		8 or more days vs. not met	2.769	0.232	<0.001	2.230	3.310
A new adult met when outside of the household (Figure 4b)	Pre-lockdown	1 to 7 days vs. 8 or more days	5.540	0.134	<0.001	5.230	5.860
		1 to 7 days vs. not met	7.460	0.260	<0.001	6.850	8.070
		8 or more days vs. not met	1.920	0.263	<0.001	1.300	2.530
	Lockdown	1 to 7 days vs. 8 or more days	4.540	0.223	<0.001	4.020	5.060
		1 to 7 days vs. not met	6.750	0.475	<0.001	5.640	7.870
		8 or more days vs. not met	2.210	0.478	<0.001	1.090	3.330
	Post-lockdown	1 to 7 days vs. 8 or more days	5.740	0.235	<0.001	5.190	6.290
		1 to 7 days vs. not met	7.760	0.477	<0.001	6.640	8.880
		8 or more days vs. not met	2.020	0.480	<0.001	0.890	3.140

**Table 5:** Pairwise comparisons for each term in the binomial regression models for the recency of socialisation experiences of 6-month-old puppies with new dogs from outside of the household, familiar dogs, and children.

Socialisation Experience	Term	Comparisons	Estimate	*SE*	*p*	95% *CI*	
						Lower	Upper
A new dog from outside of the household (Figure 4c)	Days	1 to 7 vs. 8 or more	5.030	0.116	<0.001	4.760	5.310
		1 to 7 vs. not met	7.120	0.248	<0.001	6.530	7.700
		8 or more vs. not met	2.080	0.250	<0.001	1.500	2.670
	Lockdown phase	Pre-lockdown vs. Lockdown	0.092	0.228	0.914	−0.443	0.627
		Pre-lockdown vs. Post-lockdown	−0.135	0.157	0.666	−0.502	0.232
		Lockdown vs. Post-lockdown	−0.227	0.246	0.626	−0.803	0.349
A dog that the puppy knows (Figure 4d)	Days	1 to 7 vs. 8 or more	2.590	0.075	<0.001	2.416	2.770
		1 to 7 vs. not met	3.690	0.096	<0.001	3.466	3.920
		8 or more vs. not met	1.100	0.100	<0.001	0.867	1.330
	Lockdown phase	Pre-lockdown vs. Lockdown	−0.108	0.088	0.436	−0.312	0.098
		Pre-lockdown vs. Post-lockdown	−0.036	0.081	0.894	−0.225	0.153
		Lockdown vs. Post-lockdown	0.071	0.103	0.768	−0.169	0.312
A child/children (Figure 4e)	Days	1 to 7 vs. 8 or more	2.070	0.075	<0.001	1.900	2.250
		1 to 7 vs. not met	3.640	0.111	<0.001	3.380	3.910
		8 or more vs. not met	1.570	0.113	<0.001	1.310	1.840
	Lockdown phase	Pre-lockdown vs. Lockdown	−0.005	0.104	0.999	−0.249	0.239
		Pre-lockdown vs. Post-lockdown	−0.115	0.082	0.337	−0.306	0.076
		Lockdown vs. Post-lockdown	−0.120	0.116	0.609	−0.381	0.162

## Data Availability

The data are not publicly available due to ethical approval of participant informed consent that included “Generation Pup” participants being informed that we will remove all personally identifiable information before sharing data with Universities and/or Research institutions.

## Supplementary Materials

The following supporting information can be downloaded at: https://www.mdpi.com/article/10.3390/ani14101471/s1.
