# Supplementary material for: Puppy Socialisation Experiences in Relation to Age and COVID-19 Lockdown Restrictions in the UK and ROI
Source: Animals (Basel). 2024 May 15;14(10):1471. doi: 10.3390/ani14101471 (PMC11117371; doi:10.3390/ani14101471)
Supplement: Supplementary file 1 [file animals-14-01471-s001.zip › animals-3012218-supplementary.pdf]

Supplementary Materials:

Question included in the 'Settling In', '12-week' and '16-week' questionnaires that was used to explore the types of socialisation experiences of puppies and the effect of age and the COVID-19 lockdown.

Q. In the past seven days my puppy has had the following experiences….

Note: By encountered we mean experienced/been aware of the situation, person, or animals in the question.

	Tick one box for each row		
	I'm unsure	Yes	No
Being out in public on the ground	☐	☐	☐
Being out in public in my arms	☐	☐	☐
Meeting/hearing the postman/woman	☐	☐	☐
Encountering livestock	☐	☐	☐
Being put in a stationary car or other vehicle	☐	☐	☐
Travelling in a moving car or other vehicle	☐	☐	☐
Going in the garden/yard	☐	☐	☐
Hearing loud noises (for example bangs, fireworks etc.)	☐	☐	☐
Hearing voices raised or arguments in the house	☐	☐	☐
Being near light traffic (for example cars passing, quiet road), except when inside a vehicle	☐	☐	☐
Being near heavy traffic (for example lorries, trucks passing, busy road), except when inside a vehicle	☐	☐	☐
Encountering a person riding a bicycle	☐	☐	☐
Visiting other houses than the one he/she lives in	☐	☐	☐
Having a collar put on	☐	☐	☐
Having a harness put on	☐	☐	☐
Having a lead put on	☐	☐	☐

Questions included in the '6-month' questionnaires that were used to explore the recency of socialisation experiences of puppies and the effect of the COVID-19 lockdown.

Q1. Approximately how many days is it since your puppy met a new adult he/she didn't previously know when they visited the household...

Note: By 'meeting people' we mean if your puppy was in the same room as a person and/or if outside and within approximately 3 meters/10 feet of a person.

Select one response:

- Today
- One number between 2 and 28 days
- More than 28 days
- I don't know/I can't remember
- Not applicable

Q2. Approximately how many days is it since your puppy met a new adult she didn't previously know when outside the household...

Note: By 'meeting people' we mean if your puppy was in the same room as a person and/or if outside and within approximately 3 meters/10 feet of a person.

Select one response:	
Today	
One number between 2 and 28 days	
More than 28 days	
I don’t know/I can’t remember	
Not applicable	
**Q3. In the last two months, (excluding children living in our household), how many days is it since your puppy met…**<br>*Note: By 'met' we mean children that your puppy has interacted with or been aware of whilst in close proximity to them.*	
No children	Select one response per row:<br>Today<br>One number between 2 and 28 days<br>More than 28 days<br>I don’t know/I can’t remember<br>Not applicable
A baby	
A toddler to 4 year old child	
A child or children aged 5-10 years	
A child or children aged 11-15 years	
A child or children that I couldn't guess the age/ages of	
I don’t know/can’t remember	
**Q4. Approximately how many days is it since your puppy met a new dog outside the household...**	
Select one response:	
Today	
One number between 2 and 28 days	
More than 28 days	
I don’t know/I can’t remember	
Not applicable	
**Q5. Approximately how many days is it since your puppy met a dog that she knows...**	
Select one response:	
Today	
One number between 2 and 28 days	
More than 28 days	
I don’t know/I can’t remember	
Not applicable	

Table S1. Results of the reduced beta regression model for the socialisation experiences of 8 to 19-week-old puppies. Estimates, standard error, results of significance tests (Wald’s *z* approximation), confidence intervals and the minimum and maximum of model estimates derived after excluding individuals one at a time.

Term	Estimate	*SE*	*z*	*p*	95% CI Lower	95% CI Upper	Min	Max
Intercept	0.497	0.021	23.196	< 0.001	0.458	0.537	0.494	0.499
Age: 84-105 days [1]	0.736	0.021	34.791	< 0.001	0.674	0.774	0.734	0.738
Age: 112-133 days [1]	1.072	0.022	49.365	< 0.001	1.029	1.109	1.069	1.075
Phase: Lockdown [2]	-0.059	0.031	-1.923	0.054	-0.119	0.005	-0.064	-0.054
Phase: Post-lockdown [2]	-0.070	0.026	-2.678	0.007	-0.123	-0.022	-0.073	-0.067

[1] Age: 56-77 days as reference level.
[2] Phase: Pre-lockdown as reference level.

Table S2. Results of the binomial regression model for meeting a new adult when they visited the household. Estimates, standard error, results of significance tests (Wald's *z* approximation) and confidence intervals.

Term	Estimate	*SE*	*z*	*p*	95% CI	
					Lower	Upper
Intercept	0.698	0.049	14.325	< 0.001	0.603	0.794
Phase: Lockdown [1]	-1.005	0.107	-9.351	< 0.001	-1.217	-0.795
Phase: Post-lockdown [1]	-0.231	0.093	-2.474	0.013	-0.413	-0.047
Days: 8 or more [2]	-1.492	0.069	-21.473	< 0.001	-1.629	-1.357
Days: Not met [2]	-4.537	0.167	-27.156	< 0.001	-4.880	-4.223
Lockdown × 8 or more	1.737	0.152	11.455	< 0.001	1.440	2.035
Post-lockdown × 8 or more	0.416	0.133	3.127	0.002	0.155	0.677
Lockdown × Not met	2.554	0.253	10.099	< 0.001	2.057	3.051
Post-lockdown × Not met	0.692	0.285	2.246	0.015	0.119	1.241

[1] Phase: Pre-lockdown as reference level.
[2] Days: 1 to 7 as reference level.

Table S3. Results of the binomial regression model for meeting a new adult when outside of the household. Estimates, standard error, results of significance tests (Wald's *z* approximation) and confidence intervals.

Term	Estimate	*SE*	*z*	*p*	95% CI	
					Lower	Upper
Intercept	2.695	0.092	29.393	< 0.001	2.520	2.879
Phase: Lockdown [1]	-0.486	0.179	-2.715	0.007	-0.830	-0.127
Phase: Post-lockdown [1]	0.106	0.185	0.574	0.566	-0.248	0.480
Days: 8 or more [2]	-5.542	0.134	-41.283	< 0.001	-5.811	-5.284
Days: Not met [2]	-7.458	0.260	-28.655	< 0.001	-8.004	-6.978
Lockdown × 8 or more	1.000	0.260	3.843	< 0.001	0.481	1.503
Post-lockdown × 8 or more	-0.197	0.270	-0.730	0.466	-0.739	0.323
Lockdown × Not met	0.705	0.542	1.302	0.193	-0.460	1.704
Post-lockdown × Not met	-0.301	0.543	-0.554	0.580	-1.469	0.701

[1] Phase: Pre-lockdown as reference level.
[2] Days: 1 to 7 as reference level.

Table S4. Results of the binomial regression model for meeting for meeting a new dog from outside of the household. Estimates, standard error, results of significance tests (Wald's *z* approximation) and confidence intervals.

Term	Estimate	*SE*	*z*	*p*	95% CI	
					Lower	Upper
Intercept	2.365	0.080	29.396	< 0.001	2.211	2.526
Phase: Lockdown [1]	0.090	0.189	0.476	0.634	-0.269	0.474
Phase: Post-lockdown [1]	0.148	0.165	0.898	0.369	-0.168	0.480
Days: 8 or more [2]	-4.846	0.117	-41.547	< 0.001	-5.078	-4.621
Days: Not met [2]	-7.108	0.257	-27.708	< 0.001	-7.648	-6.636
Lockdown × 8 or more	-0.156	0.273	-0.572	0.567	-0.706	0.367
Post-lockdown × 8 or more	-0.410	0.244	-1.678	0.093	-0.899	0.060
Lockdown × Not met	-0.391	0.656	-0.595	0.552	-1.888	0.770
Post-lockdown × Not met	0.369	0.434	0.851	0.394	-0.513	1.203

[1] Phase: Pre-lockdown as reference level.
[2] Days: 1 to 7 as reference level.

Table S5. Results of the binomial regression model for meeting a familiar dog (defined as a dog that has been met at least twice before). Estimates, standard error, results of significance tests (Wald's *z* approximation) and confidence intervals.

Term	Estimate	*SE*	*z*	*p*	95% CI	
					Lower	Upper
Intercept	1.158	0.054	21.561	< 0.001	1.053	1.264
Phase: Lockdown [1]	-0.164	0.118	-1.387	0.166	-0.393	0.070
Phase: Post-lockdown [1]	-0.045	0.104	-0.433	0.665	-0.248	0.161
Days: 8 or more [2]	-2.654	0.080	-33.198	< 0.001	-2.812	-2.498
Days: Not met [2]	-3.980	0.113	-35.203	< 0.001	-4.207	-3.763
Lockdown × 8 or more	0.149	0.179	0.831	0.406	-0.206	0.497
Post-lockdown × 8 or more	0.040	0.156	0.255	0.799	-0.268	0.343
Lockdown × Not met	0.664	0.225	2.953	0.003	0.216	1.099
Post-lockdown × Not met	0.205	0.212	0.964	0.335	-0.219	0.615

[1] Phase: Pre-lockdown as reference level.
[2] Days: 1 to 7 as reference level.

Table S6. Results of the binomial regression model for meeting a child/children. Estimates, standard error, results of significance tests (Wald's *z* approximation) and confidence intervals.

Term	Estimate	*SE*	*z*	*p*	95% CI	
					Lower	Upper
Intercept	0.944	0.054	17.553	< 0.001	0.840	1.051
Phase: Lockdown [1]	-0.048	0.124	-0.384	0.701	-0.288	0.198
Phase: Post-lockdown [1]	-0.145	0.105	-1.385	0.166	-0.350	0.062
Days: 8 or more [2]	-2.157	0.079	-27.399	< 0.001	-2.312	-2.003
Days: Not met [2]	-3.873	0.122	-31.619	< 0.001	-4.119	-3.638
Lockdown × 8 or more	0.099	0.181	0.546	0.585	-0.259	0.451
Post-lockdown × 8 or more	0.152	0.155	0.980	0.327	-0.154	0.454
Lockdown × Not met	0.058	0.283	0.206	0.837	-0.518	0.596
Post-lockdown × Not met	0.628	0.216	2.904	0.004	0.198	1.047

[1] Phase: Pre-lockdown as reference level.
[2] Days: 1 to 7 as reference level.
